# The Role of Early Growth Response 1 (EGR1) in Brain Plasticity and Neuropsychiatric Disorders

**DOI:** 10.3389/fnbeh.2017.00035

**Published:** 2017-03-06

**Authors:** Florian Duclot, Mohamed Kabbaj

**Affiliations:** ^1^Department of Biomedical Sciences, Florida State UniversityTallahassee, FL, USA; ^2^Program in Neuroscience, Florida State UniversityTallahassee, FL, USA

**Keywords:** early growth response 1, Zif268, synaptic plasticity, memory, stress, anxiety

## Abstract

It is now clearly established that complex interactions between genes and environment are involved in multiple aspects of neuropsychiatric disorders, from determining an individual’s vulnerability to onset, to influencing its response to therapeutic intervention. In this perspective, it appears crucial to better understand how the organism reacts to environmental stimuli and provide a coordinated and adapted response. In the central nervous system, neuronal plasticity and neurotransmission are among the major processes integrating such complex interactions between genes and environmental stimuli. In particular, immediate early genes (IEGs) are critical components of these interactions as they provide the molecular framework for a rapid and dynamic response to neuronal activity while opening the possibility for a lasting and sustained adaptation through regulation of the expression of a wide range of genes. As a result, IEGs have been tightly associated with neuronal activity as well as a variety of higher order processes within the central nervous system such as learning, memory and sensitivity to reward. The immediate early gene and transcription factor early growth response 1 (EGR1) has thus been revealed as a major mediator and regulator of synaptic plasticity and neuronal activity in both physiological and pathological conditions. In this review article, we will focus on the role of EGR1 in the central nervous system. First, we will summarize the different factors influencing its activity. Then, we will analyze the amount of data, including genome-wide, that has emerged in the recent years describing the wide variety of genes, pathways and biological functions regulated directly or indirectly by EGR1. We will thus be able to gain better insights into the mechanisms underlying EGR1’s functions in physiological neuronal activity. Finally, we will discuss and illustrate the role of EGR1 in pathological states with a particular interest in cognitive functions and neuropsychiatric disorders.

## Introduction

Despite a high level of heritability observed in the most common neuropsychiatric disorders, a clear genetic basis in their etiology has proven difficult to identify (Plomin et al., 1994). Rather, extensive evidence now indicates that genetic variations among the population markedly influence one’s vulnerability to develop neuropsychiatric disorders and thus represent major risk factors (Burmeister et al., 2008; Lee et al., 2013). Indeed, such genetic variations can underlie differences in the integration of and response to environmental insults that can transpose into deep and lasting neuroadaptations responsible for social, emotional and cognitive impairments characteristics of severe neuropsychiatric disorders (Caspi and Moffitt, 2006). In this context, it appears critical to better understand the molecular processes and mechanisms underlying such gene × environment interactions.

In the central nervous system, immediate early genes (IEGs) are critical mediators of gene × environment interactions and thus have been the focus of an extensive research interest in order to elucidate how environmental stimuli trigger a fast response with enduring neuroadaptations on neuronal activity and plasticity (Herdegen and Leah, 1998; Bahrami and Drabløs, 2016). Indeed, the defining characteristic of IEGs is the rapid and transient up-regulation—within minutes—of their mRNA levels independent of protein synthesis. Furthermore, this regulation can be triggered by a wide variety of stimuli through activation of general intracellular signaling pathways such as the mitogen-activated protein kinases (MAPK) or phosphoinositide 3-kinase (PI3K) pathways (Beckmann and Wilce, 1997; Fowler et al., 2011; Bahrami and Drabløs, 2016). Combined with the fact that many IEGs act as transcription factors, these features allow for a rapid and dynamic response to neuronal activity, followed by a second wave of transcriptional regulation likely to encode enduring adaptations at the synaptic and neuronal levels. Unsurprisingly, IEGs involvement in neuronal functions is widespread. In addition to representing key elements in understanding neuronal activity and physiological response to environmental stimuli, deciphering IEGs functions can provide a wealth of information on how these mechanisms are impaired in pathological conditions and thus bring novel insights into the molecular mechanisms underlying severe neuropsychiatric disorders.

Despite their widespread nature and overlap, each IEG differs in activators, upstream regulatory pathways, targets and expression pattern (Beckmann and Wilce, 1997; Herdegen and Leah, 1998; O’Donovan et al., 1999; Poirier et al., 2008; Bahrami and Drabløs, 2016). As such, early growth response 1 (EGR1) represents a particularly interesting IEG in the context of neuropsychiatric disorders due to its involvement in critical processes underlying neuronal activity, from neurotransmission and synaptic plasticity, to higher order processes such as learning and memory, response to emotional stress and reward. In this review aticle, we will thus focus on the role of EGR1 in the central nervous system in both physiological and pathological conditions. We will first briefly summarize the different factors regulating EGR1 expression, and then take advantage of recent genome-wide transcriptomic data to analyze the genes, pathways, and biological functions targeted by EGR1 in the central nervous system. Finally, we will discuss and illustrate the role of EGR1 in pathological states with a particular interest in cognitive functions and neuropsychiatric disorders.

## Functions and Regulations of EGR1

### Structure and Expression Pattern

EGR1 was first discovered and cloned almost three decades ago during a screening of genes rapidly up-regulated by nerve growth factor (NGF) in the rat PC12 cells in the presence of the protein synthesis inhibitor cyclohexamide (Milbrandt, 1987), thereby meeting criteria for an IEG. The same protein was cloned and described simultaneously by different groups in multiple cell lines stimulated by various growth factors, which explains the existence of several alternate names: EGR1 (Sukhatme et al., 1988), NGFI-A (Milbrandt, 1987), Krox-24 (Lemaire et al., 1988), TIS8 (Lim et al., 1987, 1989), and Zif268 (Christy et al., 1988). Notably, similar screening strategies led to the identification of EGR2, EGR3 and EGR4, which alongside EGR1 constitute the EGR family of IEGs (Beckmann and Wilce, 1997; O’Donovan et al., 1999).

**Figure 1:**
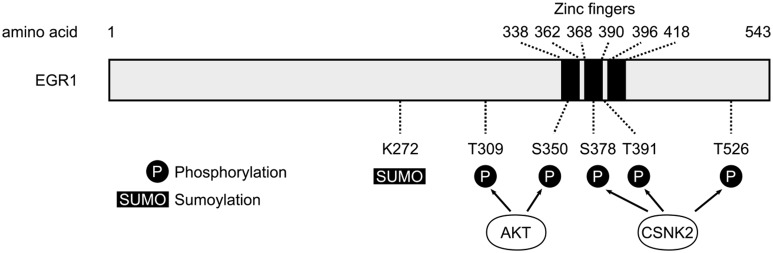
**Schematic representation of human early growth response 1 (EGR1) protein.** The three zinc fingers domains of the human EGR1 protein (Uniprot #P18146) are depicted with black bars, alongside the main post-translational modification sites identified so far (P, Phosphorylation; SUMO, Sumoylation; S, Serine; T, Threonine). The T309 and S350 sites are phosphorylated by AKT (Yu et al., 2009), whereas S378, T391 and T526 represent the main sites phosphorylated by casein kinase 2 (CSNK2; Jain et al., 1996).

The structural similarities and differences between all four EGR proteins have been described in details and summarized elsewhere (Beckmann and Wilce, 1997) and thus will not be extensively detailed in the current review article. Nevertheless, it is important to note that all four members of the EGR family are highly homologous both within and between species around a region containing three Cysteine2-Histidine2 (C_2_H_2_) zinc fingers DNA-binding domains, suggesting similarities in the DNA sequences recognized by each EGR protein and thus the possibility of overlap in their respective targets and functions (Figure 1). Similarly, EGR1, EGR2 and EGR3, but not EGR4, exhibit a domain of interaction with the transcriptional co-repressors NGFI-A-1/2 (NAB1 and NAB2) that, in addition to providing a negative control on the transcriptional activity of EGR proteins (Gashler et al., 1993; Russo et al., 1993, 1995; Svaren et al., 1996; Beckmann and Wilce, 1997), suggests that EGR1, EGR2 and EGR3 can lead to transcriptional repression—a role supported in part by experimental evidence *in vivo* (James et al., 2005, 2006; Duclot and Kabbaj, 2015). Interestingly, aligning the amino-acids sequences for all EGR proteins from humans, rats and mice, reveals that differences between EGR proteins are greater within species than between species, suggesting that similarities and specificities of each EGR member are evolutionary conserved. Despite this homology, however, the N-terminal region differs substantially between all four members of the EGR family, indicating specificities in protein-protein interactions and thus differences in regulation, reactivity, transcriptional control, and ultimately neuronal function (O’Donovan et al., 1999; Poirier et al., 2008).

In line with functional differences between members of the EGR family, the constitutive EGR2 knock-out is lethal whereas mice lacking EGR1 are viable despite reduced body size, sterility associated with alterations of the pituitary-gonadal axis, as well as axial myopia (Lee et al., 1995; Beckmann and Wilce, 1997; Topilko et al., 1998; Schippert et al., 2007), which indicates that EGR1 is not critically involved in prenatal development. Accordingly, EGR1 expression is undetectable in the embryonic nervous system (McMahon et al., 1990; Crosby et al., 1992), but slowly rises throughout postnatal development to reach adult expression levels by postnatal day 17 in the rat hippocampus, for instance (Watson and Milbrandt, 1990; Herms et al., 1994; Beckmann and Wilce, 1997). Interestingly, this progressive increase in EGR1 expression parallels the time of synaptic formation in cortical regions, and in the hippocampal CA1 area, corresponds closely to the period of maximal response to N-methyl-D-aspartate (NMDA) and long-term potentiation (LTP) inducibility (Herms et al., 1994), which underscores the relationship between EGR1 expression and synaptic plasticity. In adulthood, EGR1 is expressed widely throughout the brain, and thus maintains baseline expression levels in several key areas for control of cognition, emotional response, social behavior and sensitivity to reward such as the medial prefrontal cortex (mPFC), striatum, hippocampus and amygdala (Herdegen et al., 1995; Beckmann and Wilce, 1997; Knapska and Kaczmarek, 2004).

### Upstream Regulators

#### Signaling Pathways and Transcriptional Control

**Figure 2:**
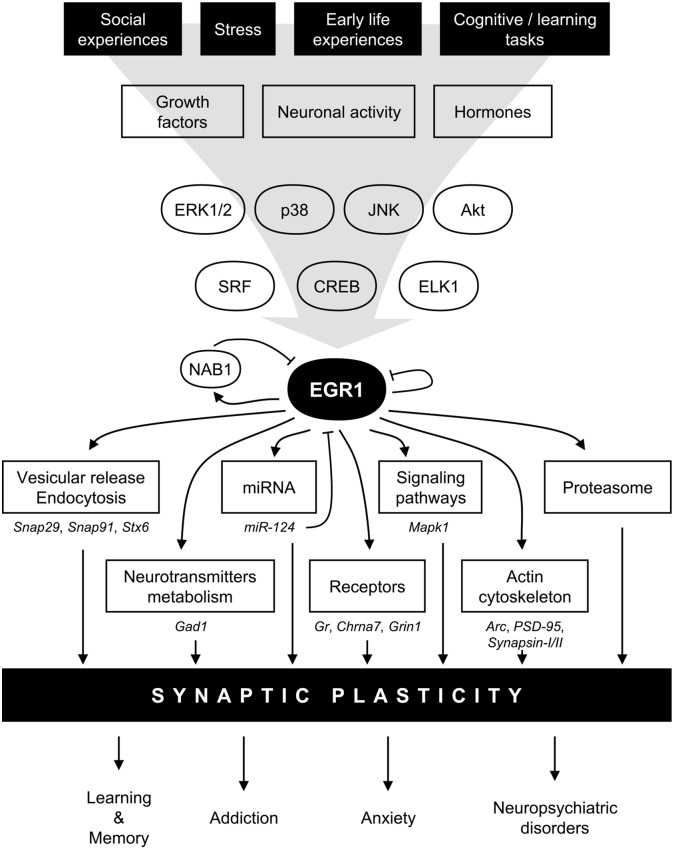
**Model for EGR1 regulations and functions in the central nervous system in the context of synaptic plasticity.** In response to various stimuli such as stress or learning tasks triggering growth factors release, hormones secretion, or neuronal activity, several intracellular signaling pathway including mitogen-activated protein kinases (MAPK) or AKT are activated. Transcription factors such as serum response factor (SRF), cyclic AMP-response element binding protein (CREB), or Ets-like-1 (ELK1), are thus induced and rapidly regulate *Egr1* transcription. EGR1 can in turn directly regulate a wide array of transcriptional targets related to multiple biological functions related to synaptic plasticity: vesicular release and endocytosis, neurotransmitters metabolism, micro-RNA (miRNA), receptors, signaling pathways, actin cytoskeleton, as well as component of the proteasome complex. A few validated EGR1 targets are depicted under each biological functions. Through such a wide array of direct transcriptional targets, EGR1 can thus regulate multiple aspects of synaptic plasticity, and thus orchestrate the integration of environmental stimuli at the synaptic plasticity level to modulate relevant high order processes such as learning and memory, addiction, anxiety, and neuropsychiatric disorders. Finally, several negative feedback mechanisms are also engaged, either directly through EGR1 itself, or indirectly through its direct targets such as NAB1 or miR-124. *Arc*, activity-regulated cytoskeleton-associated protein; *Chrna7*, cholinergic receptor nicotinic alpha 7 subunit; ERK1/2, extracellular signal-regulated kinase (ERK)s 1/2; *Gad1*, glutamate decarboxylase 1; *Gr*, Glucocorticoids receptor; *Grin1*, glutamate ionotropic receptor N-methyl-D-aspartate (NMDA) type subunit 1, JNK, Jun N-terminal kinase; *Mapk1*, mitogen activated protein kinase 1; NAB1, NGFI-A-1; *PSD-95*, Postsynaptic density protein 95; *Snap29*, synaptosomal-associated protein 29; *Snap91*, synaptosomal-associated protein 91; *Stx6*, syntaxin 6.

Following the original discovery of EGR1 induction following PC12 cells stimulation by NGF (Milbrandt, 1987), its expression levels were quickly linked to synaptic activity in mature neurons. In particular, *in vivo* electrical stimulations inducing long-term potentiation (LTP) also up-regulate *Egr1* mRNA levels in an NMDA receptor-dependent manner (Cole et al., 1989; Wisden et al., 1990). Similarly, *Egr1* mRNA levels rapidly and transiently increase in the rat forebrain, cerebellum and hippocampus following pharmacological induction of seizures (Saffen et al., 1988). Since then, the range of stimulations able to induce *Egr1* mRNA up-regulation has greatly expanded and includes a variety of factors linked to neurotransmission and synaptic activity. These include neurotransmitters such as glutamate and dopamine, their receptors such as NMDA or dopamine D1 receptors, as well as their respective agonists or cellular depolarization itself (Beckmann and Wilce, 1997; Herdegen and Leah, 1998; Knapska and Kaczmarek, 2004). In line with these extracellular signals, multiple intracellular signaling pathways downstream of these receptors directly regulate EGR1 expression. Similar to other IEGs (Bahrami and Drabløs, 2016), the RhoA-actin (Mullin et al., 2007), extracellular signal-regulated kinase (ERK; Sgambato et al., 1998; Davis et al., 2000) and p38 (Lim et al., 1998; Rolli-Derkinderen et al., 2003) MAPK, or PI3K (Kumahara et al., 1999) have been reported to control EGR1 expression in various systems, including neurons *in vivo* (Beckmann and Wilce, 1997; Herdegen and Leah, 1998; Knapska and Kaczmarek, 2004). Altogether, these observations would indicate that EGR1 expression can be activated upon a wide variety of stimuli, as reflected by its up-regulation following an intracellular calcium increase in hippocampal neurons (Bading et al., 1995), and support the notion that EGR1 is generally activated upon neuronal activity (Figure 2). While such a wide range of stimulating factors can represent a challenge in pinpointing the exact role of EGR1 in synaptic activity, this feature can be turned into an advantage by using EGR1 expression as a marker of neuronal activity allowing to map brain activation following a specific behavioral, pharmacological, or environmental event (Farivar et al., 2004; Stack et al., 2010; Okuno, 2011; Hollis et al., 2012; Duclot et al., 2016). Interestingly, EGR1’s induction following neuronal activity could also prove useful in tagging neurons activated by specific stimuli which, coupled with optogenetics, for instance, offers interesting methods to study the functions of neuronal ensembles in high order brain functions (Ramirez et al., 2015; Tonegawa et al., 2015). In this context, it is particularly interesting to note that *Egr1* promoter can successfully be used in a reporter construct (Tsai et al., 2000). Combined with the specific roles of EGR1 in regulating neuronal plasticity (see “EGR1 Role in Pathological States” Section), this provides unique opportunities to investigate the neuronal ensembles underlying anxiety, stress response, and stress-related disorders.

Upon activation, these intracellular signaling pathways will engage their respective final effector(s) and transcription factor(s) to directly regulate *Egr1* gene transcription. Induction of the p38 and ERK MAPK pathways, for instance, leads to activation of the Ets-like-1 (Elk1) and cyclic AMP-response element binding protein (CREB) transcription factors, which can bind their respective response elements located in the *Egr1* promoter (Tur et al., 2010). In addition to these serum response elements (SRE) and cAMP response element (CRE), several other binding sites for key transcription factors were identified on the *Egr1* promoter: specificity protein 1 (Sp1), activator protein-1 (AP-1), nuclear factor kappa B (NFκB), or EGR1 itself (Knapska and Kaczmarek, 2004; Tur et al., 2010). While most of these factors are generally considered as positive regulators of transcription, this view is challenged by the bivalent role of Elk1, for instance, either promoting transcription through recruitment of histone acetyltransferases (Li et al., 2003), or repressing transcription through recruitment of histone deacetylases (HDAC; Yang et al., 2001). Similarly, EGR1 binding to its own promoter represses its transcription (Cao et al., 1993).

The complexity of *Egr1* transcriptional regulation can be resolved, however, when accounting for kinetics and interactions between transcription factor binding, cofactors recruitment and chromatin dynamics including histone methylation, acetylation and phosphorylation, as well as nucleosome positioning. Indeed, by focusing on *Egr1* gene transcription in MLP29 mouse progenitor cells, Riffo-Campos et al. (2015) propose a model in which Elk1, CREB and EGR1 interact in a timely manner to allow for a quick and transient activation of *Egr1* transcription. Following application of phorbol esters in this system, EGR1 expression is induced within minutes, peaks at 30 min post-application, and returns to baseline levels by 180 min (Tur et al., 2010; Riffo-Campos et al., 2015). Prior to treatment with phorbol esters, three components of HDAC complexes, mSin3, HDAC3 and N-CoR are present on the *Egr1* promoter (Tur et al., 2010). Interestingly, however, CREB, Elk1, SRF and RNA-PolII are also found at the promoter even prior to its induction, explained in part by a favorable nucleosome positioning (Riffo-Campos et al., 2015), which thus suggests that, similar to other IEGs (Bahrami and Drabløs, 2016), *Egr1* transcription is poised at baseline. Induction by phorbol esters, however, triggers characteristic nucleosome repositioning events with partial eviction of the +1 and −1 nucleosomes, as well as downstream sliding of the −2 nucleosome at the 15 min timepoint. At the same time, *Egr1*-promoter bound CREB and Elk1 are phosphorylated in a p38- and MEK1/2-dependent manner (Tur et al., 2010), resulting in an increase in phosphoacetylation (pS10AcK14) and acetylation (AcK14) of histone H3 at the +1 nucleosome (Riffo-Campos et al., 2015). Such acetylation events are likely mediated by the histone acetyltransferase activity of the transcriptional cofactor CREB-binding protein (CBP) as its binding to the mouse *Egr1* promoter increases in parallel with its transcription (Tur et al., 2010). As a result, RNA-PolII recruitment rises and promotes *Egr1* transcription in a rapid manner. Simultaneously, however, the downstream sliding of the −2 nucleosome partly uncovers an EGR1 recognition site located slightly upstream and thus allows EGR1 binding to its own promoter (Riffo-Campos et al., 2015), which in turn leads to the progressive recruitment of the transcriptional repressors NAB1 and NAB2 peaking from 30 min–60 min following induction (Tur et al., 2010). As NAB2 is known to interact with the nucleosome remodeling and deacetylase complex (NuRD; Srinivasan et al., 2006), it is likely that this interaction is responsible for the progressive decline in histone acetylation and phosphoacetylation, as well as the return of nucleosomes to baseline positions leading to reduction in EGR1 expression (Tur et al., 2010; Riffo-Campos et al., 2015). Interestingly, NAB2 is not constitutively expressed but induced by factors such as EGR1, which does provide a negative feedback loop mechanism for EGR1 expression allowing to explain the transient nature of its expression.

#### Epigenetics, Post-Translational Modifications and Other Regulators

Importantly, such regulations of *Egr1* transcription by histone acetylation and methylation events are also found in neurons *in vivo* as part of neuroadaptations underlying learning and memory, cognitive functions and response to stress (Gräff et al., 2012; Xie et al., 2013; Hendrickx et al., 2014; Rusconi et al., 2016). Furthermore, DNA methylation and hydroxymethylation have also been linked to the control of *Egr1* transcription associated with environmental impact on synaptic transmission upon aging in the rat hippocampus (Penner et al., 2016), or sleep deprivation in the mouse cortex (Massart et al., 2014). Altogether, it is therefore clear that epigenetic mechanisms are not only an essential part of *Egr1* regulation, but also key mediators of neuroadaptations critical to physiological and pathological brain functions.

Furthermore, EGR1 levels can be regulated on another epigenetic layer through micro-RNA (miRNA). Indeed, in peripheral tissues and several cancer cell lines, several studies report direct targeting of EGR1 by miR-543 (Zhu et al., 2016), miR-192 (Wu et al., 2016), miR-146a (Contreras et al., 2015), miR-7578 (Zhang et al., 2013), miR-183 (Sarver et al., 2010), or miR-124 (Liu et al., 2016; Wang et al., 2016). Interestingly, the latter is highly expressed in the brain and is a critical regulator of neuronal function and thus an important mediator of neuroadaptations in response to chronic stress, reward and learning and memory (Sun et al., 2015). In line with the involvement of EGR1 in these processes as well, a regulation of EGR1 levels by miR-124 has also been reported in the central nervous system. Indeed, miR-124 knockdown in the mouse mPFC and hippocampus increase EGR1 mRNA and protein levels, reflected by improvements in spatial learning and social behaviors impaired in exchange protein directly activated by cyclic AMP (EPAC)-knockout (KO) mice (Yang et al., 2012). Interestingly, although no effect on synaptic transmission was observed, this effect was associated with complete restoration of LTP that was previously abolished in EPAC-KO mice (Yang et al., 2012), which thus indicates that EGR1-targeting miRNA are likely to be involved in the numerous functions under control of EGR1.

In addition to such epigenetic mechanisms, EGR1 transcriptional activity or stability can also be dynamically regulated through post-translational modifications (Figure 1) including phosphorylation, acetylation, sumoylation and ubiquitination (Beckmann and Wilce, 1997; Veyrac et al., 2014). For instance, while EGR1 phosphorylation levels are very low in unstimulated cells, EGR1 proteins induced by growth factors or UV radiations undergo substantial phosphorylation events—involving in part protein kinase C or tyrosine kinases—resulting in an increase in its DNA binding activity (Cao et al., 1992, 1993; Huang et al., 1998). Similarly, EGR1 can be acetylated by the histone acetyltransferase complex p300/CBP, which reduces its transcriptional activity (Yu et al., 2004). Interestingly, EGR1 can undergo sumoylation and ubiquitination, and has been reported to interact directly with proteasome component C8, describing a likely mechanism controlling its targeting for proteolysis by the ubiquitin-dependent proteasome pathway (Bae et al., 2002; Li et al., 2015). Notably, such regulation has been observed following stimulation of ECV304 cells by epidermal growth factor, which increases sumoylation and ubiquitination levels of endogenous EGR1 proteins, ultimately leading to higher EGR1 turnover through proteasome-mediated degradation (Manente et al., 2011). Altogether, these observations suggest that post-translational modifications are critical regulators of EGR1 activity and stability. As further illustration, a detailed mechanistic work describes a signaling pathway in which EGR1 is phosphorylated at the T309 and S350 residues by Akt in response to insulin-like growth factor 1, thereby enhancing its interaction with alternate reading frame (ARF) which mediates sumoylation of EGR1 at the K272 residue and activation of the protein phosphatase and tensin homolog (PTEN; Yu et al., 2009). As such modifications can be observed in the brain following cocaine exposure, for instance (Xu and Kang, 2014), post-translational modifications thus represent a critical level in the regulation of EGR1 functions in the central nervous system.

Finally, it is important to note that EGR1 expression differs between strains (Pollak et al., 2005) and sexes in the central nervous system, in a structure-specific manner. Indeed, adult female rats exhibit lower EGR1 mRNA and protein levels than males in the mPFC, but not in the striatum, or hippocampal CA1 area (Stack et al., 2010; Duclot and Kabbaj, 2015; Yagi et al., 2016). Interestingly, the sex bias is opposite in the dorsal CA3 area, where the density of EGR1-expressing cells is higher in female rats than males (Yagi et al., 2016). A possibility to explain such sex differences in EGR1 expression could reside in the ovarian hormone estrogen, as the latter can directly up-regulate EGR1 expression. In the mouse mammary gland, for instance, EGR1 is at the center of a gene regulation network triggered by exposure to estrogen (Lu et al., 2008), while its mRNA levels in the mouse uterus are up-regulated following estrogen treatment (Kim et al., 2014). Surprisingly, although an estrogen response element (ERE) has been identified on the *Egr1* promoter, the induction of *Egr1* transcription by estrogen is mediated by SRF and Elk1 binding to SRE rather than binding of estrogen receptors to their ERE, and is blocked by a MAPK but not PI3K pathway inhibitor in rat cardiomyocytes or MCF-7 human breast cancer cells (Slade and Carter, 2000; Chen et al., 2004), indicating that EGR1 is a downstream target of estrogen’s non-genomic effects. Interestingly, treatment with progesterone either doesn’t affect *Egr1* mRNA (Lu et al., 2008), or dampens the estrogen-induced up-regulation of *Egr1* mRNA in the mouse uterus (Kim et al., 2014), which suggests that ovarian hormones can interact to regulate EGR1 expression. These interactions are likely to be specific to neurons, however, as Egr1, among other IEGs, is strongly up-regulated in Schwann cells following progesterone treatment (Mercier et al., 2001). Accordingly, we recently found that *Egr1* mRNA levels in the rat mPFC vary across the estrous cycle with lower levels in the early afternoon of proestrus than in diestrus (Duclot and Kabbaj, 2015), which therefore opens the possibility that genes and biological pathways under direct control of EGR1 also differ between sexes in an estrous cycle-dependent manner.

### Downstream Targets

Inherent from the characteristic features of an IEG, EGR1 is rapidly up-regulated in neurons following neuronal activity and orchestrates a subsequent wave of gene regulation to allow for the long-term and enduring encoding of the neuronal information. Surprisingly, despite its well-known association with several processes of neuronal and synaptic plasticity, the precise mechanisms by which EGR1 influences these processes remains unclear. In particular, relatively little is known as to its exact transcriptional targets and gene expression profile under its control, especially in a neuronal context.

From its original cloning nearly three decades ago and the description of three zinc fingers binding domains, the 9-nucleotide long sequence GCGG/TGGGCG was defined as the EGR1 recognition sequence (Christy and Nathans, 1989; Pavletich and Pabo, 1991). The presence of this specific EGR response element could thus theoretically be a good indicator of a direct transcriptional control by EGR1. Nevertheless, a more detailed analysis of EGR1 binding sequence revealed variation in this sequence and identified an optimal site of at least 10 nucleotides rather than 9 (Swirnoff and Milbrandt, 1995). Moreover, experimental evidence indicates that EGR1 can also regulate gene expression through interaction with other transcription factors such as c/EBPβ, Fos, or Jun (Levkovitz and Baraban, 2002; Zhang et al., 2003; Knapska and Kaczmarek, 2004; Cheval et al., 2012), which thus further expands the range of potential EGR1 targets and related biological pathways under its control.

The investigation of EGR1 targets was first conducted on a single-gene basis, through the focus on a particular cellular regulation in a given system. Although this approach led to the identification of numerous EGR1 target genes (Beckmann and Wilce, 1997; Herdegen and Leah, 1998; Knapska and Kaczmarek, 2004), the vast majority of EGR1 potential targets remained to be deciphered. In the early 2000s, the popularization of genome-wide techniques opened the possibility to search for EGR1-regulated genes on a large scale. In several prostate carcinoma cell lines, in which EGR1 is found overexpressed, endogenous or adenovirus-mediated overexpression of EGR1 impacts the expression of multiple genes, including several growth factors such as insulin-like growth factor II (Igf2), platelet-derived growth factor-A (PDGF-A), and transforming growth factor-β1 (TGF-β), as well as membrane-associated proteins, transcription factors and cofactors, all strengthening the involvement of EGR1 in response to growth factors, tumor progression, and apoptosis in these systems (Svaren et al., 2000; Virolle et al., 2003; Arora et al., 2008). Notably, the largest gene class identified in the prostate carcinoma cell lines following EGR1 overexpression includes several neuroendocrine-related genes found highly expressed in the central nervous system (Svaren et al., 2000), which pinpoints a direct control of neuron-specific genes by EGR1. It is important to note, however, that these regulations are in part cell-specific as similar microarray analyses in human endothelial cells overexpressing EGR1 revealed a different gene regulation profile despite common targets such as TGF-β, Igf2, and p57^kip2^ (Fu et al., 2003). Furthermore, a recent investigation of miRNA directly regulated by EGR1 in the human erythroleukemia cell line K562 reported a total of 124 distinct miRNA and 63 pre-miRNA bound by EGR1 following stimulation by phorbol ester—which activates EGR1 expression in this cell line (Wang et al., 2010). One of these miRNA, miR-124, is of particular interest as it is a known regulator of EGR1 levels (Liu et al., 2016; Wang et al., 2016), including in the central nervous system (Yang et al., 2012), and is tightly associated with neuronal function and higher order processes (Sun et al., 2015). Therefore, in addition to represent a likely mediator in EGR1 control of neuronal activity, these observations suggest that miR-124 could be involved in a negative feedback loop controlling EGR1 expression at the post-transcriptional level.

In order to better characterize how EGR1 binds to its target genes to regulate their transcription, and in an effort to better predict the potential direct EGR1 targets, several studies have investigated EGR1 binding through chromatin immunoprecipitation (ChIP) following by microarray profiling in monocytic differentiation of human monoblastoma cells or following UV-induced apoptosis in prostate carcinoma cells (Arora et al., 2008; Kubosaki et al., 2009). While these studies provide rich information regarding their specific systems, a more comprehensive understanding of EGR1 binding can be drawn from the effort of the Encyclopedia of DNA Elements (ENCODE) project. Indeed, as the ENCODE project included EGR1 as part of the tier 1 chromatin immunoprecipitation followed by deep sequencing (ChIP-seq), a wealth of information regarding EGR1 DNA binding characteristics and target genes has been made available (ENCODE Project Consortium, 2012). In particular, we are thus able to analyze and compare the binding pattern of 161 transcription factors across 91 cell types and a total of 4,380,444 genomic regions, among which 44,985 correspond to an EGR1 binding event. Out of the 15,872 genes thus annotated, 8552 (53.9%) contain at least one EGR1 binding region (peak) within 3 kb of their transcription start site (TSS), which indicates that across several human cell types, EGR1 can bind a very large number of genes and thus potentially regulate a very large gene expression profile (see full annotated list in Supplementary Table S1). As previously reported, EGR1 binds in close vicinity to the TSS (Project Kubosaki et al., 2009; ENCODE Project Consortium, 2012), but even though 41.6% of all EGR1 peaks are located within the promoter region, 26.4% are located within intronic regions. Notably, in line with the high GC content in the EGR1 consensus binding sequence, 31% of all annotated EGR1 peaks are located within a known CpG island, as previously reported by ChIP-chip promoter array analysis in human monoblastoma cells under monocytic differentiation (Kubosaki et al., 2009). Pending further analysis of CpG island and DNA methylation, the presence of a CpG island would thus appear to be a useful informative feature refining the prediction of putative EGR1 binding to a given gene across a variety of cell types.

**Figure 3:**
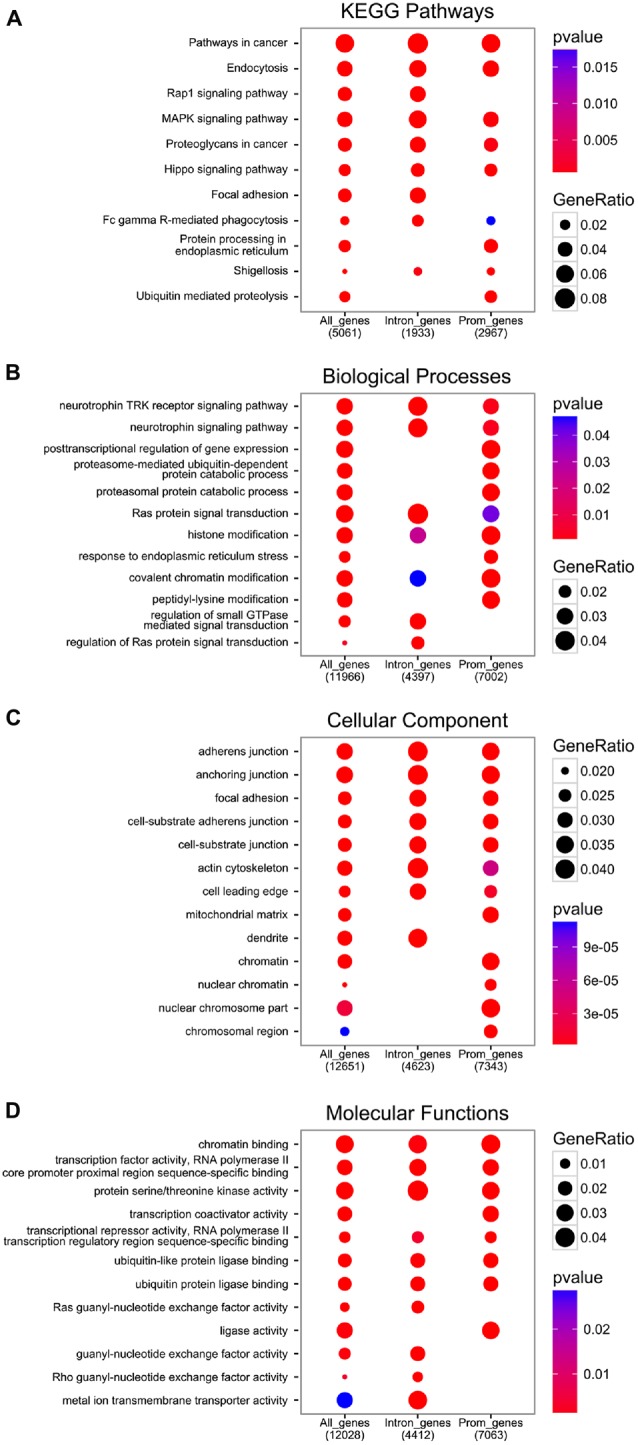
**Functional analysis of EGR1 targets from the encyclopedia of dna elements (ENCODE) datasets.** All genes annotated near an EGR1 peak (“All_genes”), or those with at least one EGR1 peak called within their promoter region (3 kb around transcription start site (TSS), “Prom_genes”), or within their intronic region (“Intron_genes”), were functionally annotated with the Kyoto Encyclopedia of Genes and Genomes (KEGG) database **(A)** and the gene ontology database distinguishing between the biological processes **(B)** cellular component **(C)** and molecular functions **(D)** domains with the Bioconductor package ChIPSeeker (v1.8.9; Yu et al., 2015).

The functional analysis of genes with at least one EGR1 peak from the ENCODE dataset reveals the enrichment of pathways and processes related to growth factors signaling, including neurotrophins, as well as general intracellular signaling cascades such as Ras or MAPK, which also controls EGR1 expression itself (Figure 3, and “Upstream Regulators” Section). Interestingly, the molecular functions of EGR1-bound genes range from chromatin and transcription factors activity to guanyl-nucleotide exchange factor activity through serine/threonine kinase activity (Figure 3D), which therefore indicates that EGR1 exerts a transcriptional control on every level of signal transduction cascade, from second messenger to transcription factor. Accordingly, the cellular localization of the EGR1-bound genes’ products range from the chromatin to the cell membrane (Figure 3C). It is important to note, however, that the latter encompasses the top enrichment hits, and reflects an enrichment of a large number of processes and pathways related to cell-cell recognition and interactions, observed across all enrichment domains (Figure 3), which suggests that EGR1 is likely to regulate cell-cell communication through a wide number of genes. Although this observation emerges from non-neuronal cell types (ENCODE Project Consortium, 2012), similar observations were made in the mouse brain. Indeed, following EGR1 ChIP-seq in the mouse cortex, a total of 11,103 genes were found bound by EGR1 in close vicinity to their TSS and were enriched for biological processes and pathways related to protein trafficking, synaptic vesicles transport, endocytosis, protein phosphorylation and intracellular signaling cascades (Koldamova et al., 2014). In this context, the relations of EGR1-bound genes with multiple levels of cell-cell communication, from reorganization of the actin cytoskeleton, to transcription factors through intracellular signaling cascades grant EGR1 the ability to control neuronal activity in a widespread manner.

In addition to the biological functions described above, a distinct pattern associated with EGR1-bound genes relates to proteasome-mediated and ubiquitin-dependent protein degradation, found in all annotation domains analyzed (Figure 3). Interestingly, while the regulation of growth factors signaling and transcription factors-related processes were observed in genes bound by EGR1 in their promoter or intronic regions, the enrichment of proteasome-mediated degradation processes preferentially involves genes bound by EGR1 in intronic regions (Figure 3B). Although its functional significance remains unknown, this may indicate that EGR1 control of proteasomal degradation-related genes is mediated through binding to enhancer regions or alternative TSS. Most importantly, such link between EGR1 and proteasome-mediated protein degradation is also found in neurons, as viral overexpression of EGR1 in cultured neuronal PC12 cells affects the expression of 135 genes, enriched for components of the proteasome and ubiquitin-related factors (James et al., 2005). Similarly, transgenic overexpression of EGR1 in the mouse forebrain results, in the amygdala, in the up-regulation of proteins related to the proteasome-core complex, among other processes such as metabolism, phosphorylation, or metal ion transport (Baumgärtel et al., 2009). Given that these regulations were also associated with genes involved in intracellular signaling, synapse formation and architecture, as well as neurotransmitter release, it is tempting to conclude that EGR1 is a master regulator of neuronal activity at multiple level of the synaptic and neuronal plasticity processes by orchestrating a widespread gene expression profile (Figures 2, 3).

It is important to keep in mind, however, that although the majority of genes affected by EGR1 overexpression present with one or more predicted EGR response elements (James et al., 2005; Baumgärtel et al., 2009), the absence of direct measurement of EGR1 binding and the possibility of detecting extra-physiological EGR1 transcriptional activity due to exogenous overexpression cannot be ruled out. Recently, however, we took advantage of endogenous differences in EGR1 expression levels in the rat mPFC between males and females, as well as within females across the estrous cycle, to provide additional *in vivo* ChIP-seq information on its direct targets underlying its role in neuronal activity. We thus found that between proestrus and diestrus females, the transcriptomic changes were very large and paralleled by widespread differential binding of EGR1 throughout the genome (Duclot and Kabbaj, 2015). Supporting James et al. (2005) findings in cultured neuronal cells, the EGR1-bound genes were highly enriched for biological processes related to synaptic function—neurotransmitters, signal transduction, presynaptic vesicular trafficking, synapse formation and assembly, and protein translation and degradation (Duclot and Kabbaj, 2015). Notably, this enrichment was even stronger when considering only the genes detected by RNA-seq as differentially expressed, which strongly suggests that EGR1-binding to these genes was transcriptionally effective.

## EGR1 Role in Synaptic Plasticity

Following the original report of *Egr1* mRNA levels increase by NGF (Milbrandt, 1987), stimulation of neuronal activity was soon identified as a potent trigger for EGR1 induction. In particular, high, but not low, frequency stimulation of the perforant path, which induces LTP, increased *Egr1* mRNA levels in the ipsilateral granule cell neurons (Cole et al., 1989). Importantly, NMDA receptors antagonism or simultaneous synaptic inputs inhibiting LTP were able to block this response, and thereby were the first demonstration that EGR1 expression can be induced by conditions favorable to LTP formation. At the molecular level, this EGR1 regulation requires the MAPK MEK and triggers the ERK1/2, Elk1 and CREB signaling cascade (Davis et al., 2000; Veyrac et al., 2014). Interestingly, early correlations between EGR1 levels and LTP expression pointed towards a link between EGR1 and LTP persistence, rather than its induction (Richardson et al., 1992; Abraham et al., 1993; Knapska and Kaczmarek, 2004). This was confirmed later in EGR1-KO mice in which early hippocampal LTP was intact, but was not present 24–48 h post-tetanic stimulation while, indicating that EGR1 is required specifically for the maintenance of LTP, but not its induction (Jones et al., 2001). Conversely, EGR1 overexpression in the forebrain enhances LTP in the mouse dentate gyrus (Penke et al., 2014). As hippocampal LTP is considered a molecular hallmark of spatial memory formation (Sweatt, 2016), the role of EGR1 in learning and memory paradigms has been extensively studied and recently reviewed elsewhere (Bozon et al., 2002; Knapska and Kaczmarek, 2004; Veyrac et al., 2014). This will thus not be discussed in detail here.

It is important to note, however, that in line with the important role of EGR1 in late-phase LTP, short-term spatial memory is intact in EGR1-KO mice, while spatial long-term memory is impaired (Jones et al., 2001), suggesting a critical role for EGR1 in memory consolidation. Although EGR1 is up-regulated following a wide range of learning procedures, this effect remains structure-specific and is generally observed in the brain regions relevant to the nature of the learning task (Veyrac et al., 2014), in line with its induction by neuronal activity. Moreover, the functional and behavioral outcome of EGR1 up-regulation in learning in memory is also specific to the nature of the task. For instance, although EGR1 knockdown by RNA interference in the amygdala impairs the consolidation of cued and contextual fear memory, EGR1 knockdown in the hippocampus impairs contextual memory reconsolidation but not consolidation—in line with the known distinction in molecular events recruited under memory consolidation and reconsolidation (Lee et al., 2004; Veyrac et al., 2014). Notably, recent evidence derived from RNA interference experiments in rats suggest that EGR1’s role in memory reconsolidation rather reflects suppression of extinction upon short memory recall and thus tilting of the balance between activation of extinction or reconsolidation towards the latter (Trent et al., 2015). Interestingly, EGR1 involvement may not be restricted to memory encoding but is likely to be expanded to neuronal encoding in a more global way. Indeed EGR1 deletion in mice destabilizes the spatial representation of a familiar environment in hippocampal CA1 place cells, and impairs the long-term, but not the short-term stabilization of a novel environment (Renaudineau et al., 2009). In the same cells, EGR1 is up-regulated during a water maze procedure regardless of the memory performance or even in a non-learning version of the task, which suggests that EGR1 up-regulation in place cells is activated each time the animal enters an area related to the given place cells and thus reflects spatial encoding rather than memory encoding (Rapp et al., 1987; Guzowski et al., 2001; Shires and Aggleton, 2008; Laeremans et al., 2015), in line with the functional role played by this cell population (O’Keefe and Dostrovsky, 1971). Similarly, Carter et al. (2015) recently observed that exposure to a water maze task increases EGR1 and c-Fos expression through ERK1/2 activation and histone H3 phosphoacetylation throughout the rat hippocampus regardless of the learning component, although the effects were most pronounced in the dentate gyrus.

Notably, while EGR1 regulation by neuronal activity and plasticity underlying memory processes are well documented, the exact transcriptional targets involved remain unclear. Under this perspective, it is particularly interesting to consider another IEG: Arg3.1 (also known as ARC). Indeed, EGR1 binds to *Arc* promoter *in vivo* following synaptic activation and triggers its transcription (Li et al., 2005). On a functional level, ARC shares a lot of similarities with EGR1. Indeed, ARC is an IEG up-regulated in neurons following synaptic activity, is involved in the maintenance of LTP, and is required for long-term memory consolidation but not short-term memory formation or learning (Minatohara et al., 2015)*.* Contrary to EGR1, however, ARC mRNA and proteins can be found in dendrites and post-synaptic locations (Kobayashi et al., 2005) where it is believed to function by interacting with other post-synaptic proteins. In particular, ARC interacts with endophilin and dynamin to enhance endocytosis of α-amino-3-hydroxy-5-methyl-4-isoxazolepropionic acid (AMPA) receptors, but also interacts with the actin cytoskeleton in dendritic spines where it is required for cofilin phosphorylation and local F-actin expansion (Chowdhury et al., 2006; Bramham et al., 2008). As both processes are critical underpinning of major synaptic plasticity events such as LTP, ARC-mediated reorganization of actin cytoskeleton and synaptic architecture represents a very promising candidate in mediating EGR1’s critical role in synaptic plasticity and related behavioral outcomes such as memory consolidation. It is important to note, however, that ARC can also be regulated independently of EGR1, as observed following intra-hippocampal brain-derived neurotrophic factor infusion in rats (Ying et al., 2002), which, in addition to illustrating the diversity in ARC regulation, further illustrates the specificity in EGR1 recruitment underlying neuronal and synaptic plasticity.

Interestingly, recent genome-wide investigations of EGR1 transcriptional targets point towards a widespread regulation of genes associated with similar dynamics critical in regulating synaptic plasticity. Indeed, a multitude of genes related to vesicular transport and neurotransmitter release, clathrin-dependent endocytosis (involved in post-synaptic receptor internalization), or actin cytoskeleton, are commonly observed as direct EGR1 targets (ENCODE Project Consortium, 2012; Koldamova et al., 2014; Duclot and Kabbaj, 2015), suggesting that besides ARC, many other EGR1 targets related to these processes may be involved in the regulation of synaptic activity by EGR1. In this context, it is particularly interesting to note that EGR1 was recently described as recruited to the postsynaptic density 95 (PSD-95) gene promoter in response to NMDA receptor activation in hippocampal primary neurons, leading to its repression (Qin et al., 2015). As a result, EGR1 knockdown in rat hippocampal neurons blocks NMDA receptors-induced PSD-95 down-regulation and AMPA receptor endocytosis, while its overexpression has the opposite effects (Qin et al., 2015). Similarly, the observation that EGR1 controls the expression of genes related to protein translation and ubiquitin-dependent degradation (James et al., 2005, 2006; Baumgärtel et al., 2009) indicates that EGR1 can coordinate a complex transcriptional program leading to a synaptic reorganization at multiple levels that promotes stabilization of the synapse, which would be in line with the repeated involvement of this IEG in encoding synaptic information.

## EGR1 Role in Pathological States

As described above, EGR1 is regulated by a wide variety of environmental stimuli and can regulate a large transcriptional program related to critical processes underlying synaptic plasticity and encoding of information. As a result, EGR1 represents a key factor both in integrating perception of the environment and in shaping an appropriate response. In this context, it is therefore not surprising to find EGR1 associated with neuropsychiatric illnesses in which neuronal plasticity and activity is altered or dysfunctional. In the sub-sections below, we will thus focus on some of the main neuropsychiatric disorders in which EGR1 has been implicated.

### Response to Stress

Despite their high prevalence (Kessler et al., 2012), stress-related mood disorders such as anxiety and depression still remain elusive in their exact etiology. Nevertheless, repeated exposure to stressful experiences is now established to represent one of the main risk factors for their development. As a result, a multitude of animal models for depression and anxiety disorders relying on the repeated exposure to stress of different nature have been developed (Czéh et al., 2016). In this context, it is important to first better understand EGR1’s regulation and role in response to stress and in such animal models.

In accordance with its activation by neuronal activity, EGR1’s regulation following exposure to stress is variable depending on the nature and duration of the stress. An acute physical stressor, such as restraint, immobilization, or forced swim, leads to increase in *Egr1* mRNA levels throughout the brain including neocortical areas, hippocampus, lateral septum, caudate putamen, nucleus accumbens, amygdala, and paraventricular nucleus (PVN) of the hypothalamus (Schreiber et al., 1991; Melia et al., 1994; Watanabe et al., 1994; Cullinan et al., 1995; Olsson et al., 1997; Knapska and Kaczmarek, 2004; Kozlovsky et al., 2009). Despite such a strong response to an acute stress, repetition of the same stress blunts the stress-induced EGR1 response, as observed in the PVN, hippocampus, or cortical regions (Melia et al., 1994; Watanabe et al., 1994; Girotti et al., 2006), and denotes a physiological habituation to homotypic exposures to physical stressors. Despite this habituation, however, exposure to a novel stress (shaking stress vs. restraint stress) still leads to a full increase in *Egr1* mRNA levels in the PVN (Watanabe et al., 1994), which thus indicates that habituation to the stressor is stress-specific. Nevertheless, one study investigating the effects of immobilization stress on IEGs expression in the PVN found that whereas c-Fos and EGR1, among others, are up-regulated upon acute immobilization, repetition of this stress for 6 days suppresses such response for c-Fos, but not for EGR1 (Umemoto et al., 1994, 1997). Furthermore, as chronic treatment with high concentration of glucocorticoids (corticosterone) mimics the effects of repeated stress exposure, the authors concluded that glucocorticoids mediate the habituation of IEGs in the PVN to repeated stress exposure while EGR1 was resistant to such effect (Umemoto et al., 1997). As EGR1 is a reliable marker of neuronal activity, these findings can be seen as illustrations of the importance of the nature and intensity of the stress on the neuronal and transcriptional response and habituation upon repeated exposure. Interestingly, although outside of the central nervous system, a recent genome-wide investigation in the rat adrenal medulla of the evolution of genes whose regulation is correlated with EGR1 following one or six exposures to immobilization stress revealed a distinct profile of interactions across time. Indeed, while EGR1 is up-regulated in the rat medulla following both acute (1×) and repeated (6×) exposure to the immobilization stress (Liu et al., 2008), its gene interaction network differed between the two stress conditions, indicating that EGR1 has different targets and functions between acute and repeated exposure to an immobilization stress (Papanikolaou et al., 2014). Altogether, stress is a major trigger for EGR1 induction in the central nervous system. Nevertheless, this activation mostly reflects the pattern of neuronal activity in response to various stressors and, as a result, varies with the nature, duration and intensity of the stress. This point is particularly well illustrated by the positive correlation observed between the magnitude of HPA axis activity, as measured by plasma adrenocorticotropic hormone (ACTH) levels, and *Egr1* mRNA levels in the PVN—an intrinsic component of the hypothalamic-pituitary-adrenals (HPA) axis—but not in the hippocampus or cortical regions for which EGR1 signal was more related to the exploration of the environment (Pace et al., 2005).

Notably, in addition to being regulated by exposure to stressful experiences, evidence indicates that EGR1 is a critical factor in encoding the behavioral enduring effects of stress. Indeed, acute exposure to forced swim stress or activation of the glucocorticoid receptor (GR) up-regulates EGR1 expression in the rat or mouse hippocampus, which mediates stress-related fear memories (Revest et al., 2005, 2010; Saunderson et al., 2016). Interestingly, such stress-induced EGR1 up-regulation depends on the methylation status of its promoter (Saunderson et al., 2016) and results in an increase in the expression and activation of MAPK pathway-associated proteins (Revest et al., 2005) as well as the synaptic plasticity-associated protein synapsin-I (Revest et al., 2010). Combined with the blockade of stress-related fear memory or GR-induced synapsin-I expression in these paradigms by synapsin-I or EGR1 knockdown, respectively, these data support a model in which EGR1 expression in the rodent hippocampus is highly regulated by stress exposure, and in turn controls synapsin-I expression to influence the synaptic plasticity underlying the consolidation of stress-related memory (Revest et al., 2010).

### Stress-Related Mood Disorders and Schizophrenia

Such variability in EGR1 response depending on the nature of the stress is also particularly important in understanding the link between EGR1 and the behavioral outcome of stress. As a result, in postmortem tissue from patients suffering from major depressive disorder, in which stress is a major risk factor (Czéh et al., 2016), EGR1 levels in the prefrontal cortex are lower when compared to healthy controls (Covington et al., 2010). Notably, such reduction was observed in both unmedicated and medicated subjects not responding to treatment and thus suggests that EGR1 levels in the mPFC are directly associated with a depressive phenotype and could be seen as a marker or mediator of positive response to antidepressant treatment (Covington et al., 2010). In light of the tight link between EGR1 expression and neuronal plasticity, the down-regulation of EGR1 in the PFC of depressed patients is particularly interesting and could represent one of the substrates for the anatomical and functional alterations observed in major depressive disorders in this brain area (Krishnan and Nestler, 2008; Koenigs and Grafman, 2009; Lefaucheur et al., 2017). In this context, a similar dysregulation of EGR1 expression is observed in other neuropsychiatric disorder characterized by functional alterations in PFC activity such as schizophrenia, where *Egr1* mRNA levels are also found down-regulated in the dorsolateral prefrontal cortex (Yamada et al., 2007; Kimoto et al., 2014). Interestingly, EGR1 levels in the PFC of schizophrenia patients are positively correlated with the mRNA levels for the glutamic acid decarboxylase 1 (GAD1), whose down-regulation is a robust molecular feature of schizophrenia subjects (Pérez-Santiago et al., 2012; Kimoto et al., 2014). Notably, although other IEGs such as c-Fos, c-jun, or EGR2 are also altered in schizophrenia, their expression levels are not correlated with GAD1 mRNA levels (Kimoto et al., 2014), which thus supports a specific role for EGR1 in GAD1 regulation and highlights its function as an important factor in the altered cortical GABA synthesis and cognitive functions observed in this neuropsychiatric disorder. Finally, in the search for schizophrenia biomarkers in peripheral tissues, EGR1 levels in whole blood samples was associated with schizophrenic symptoms such as high delusional states (Kurian et al., 2011). Similarly, EGR1 was among six genes identified as up-regulated in fibroblasts from schizophrenic patients, and the only one confirmed in peripheral blood cells as well (Cattane et al., 2015). Although this regulation is opposite to the down-regulation observed in the dorsolateral prefrontal cortex of schizophrenia patients (Kimoto et al., 2014), the up-regulation of EGR1 in blood cells was specific for schizophrenia when compared to major depressive disorder or bipolar disorder (Cattane et al., 2015), which confers EGR1 a particularly promising biomarker potential in a clinical environment. Altogether, these findings support a role for EGR1 in both the etiology and therapeutic interventions in schizophrenia.

In line with these clinical observations, *Egr1* mRNA levels are generally found down-regulated in specific brain areas in animal models inducing depressive- and anxiety-like states. For instance, the exposure of male mice to 14 days of chronic unpredictable stress leads to reduced levels of *Egr1* mRNA in the hippocampus associated with cognitive impairments in a water maze learning, novel object recognition and location tasks, CA1 basal dendrites atrophy, and altered ERK1/2 phosphorylation (Xu et al., 2015). Similarly, while an acute social defeat stress increases *Egr1* mRNA in the male mouse hippocampus (Rusconi et al., 2016), reduced *Egr1* mRNA levels in the mouse mPFC are found following repeated social defeat (Covington et al., 2010), a well-established animal model for depressive- and anxiety-like states (Hollis and Kabbaj, 2014). Notably, EGR1 expression is also reduced in the prefrontal cortex of human depressed subjects unmedicated or not responding to treatment, which thus suggests that EGR1 levels in the mPFC are directly associated with a depressive phenotype and could be seen as marker or a mediator of positive response to antidepressant treatment (Covington et al., 2010). Accordingly, reduced *Egr1* mRNA levels in the brain are commonly observed in another well-established animal model of depressive-like state, social isolation. Indeed, reduced EGR1 expression is observed in the PVN, mPFC, HPC, or extended amygdala of rats, mice and prairie voles following social isolation (Northcutt and Lonstein, 2009; Matsumoto et al., 2012; Hodges et al., 2014; Okada et al., 2014, 2015; Hodges and McCormick, 2015; Ieraci et al., 2016).

Despite the strong association of EGR1 expression levels with depression- and anxiety-like behaviors described above, the evidence for a functional link was obtained from the behavioral phenotype of EGR1-KO mice, which present with lower anxiety levels reflected by higher exploratory behavior in the open arms of an elevated plus maze (Ko et al., 2005). Since then, the role of EGR1 in regulating anxiety has been further described and targeted to the mPFC, although other structures such as the amygdala or ventral HPC are likely to contribute. In particular, we demonstrated that EGR1 expression levels in the rat mPFC control the social interaction behavior, an indicator of social anxiety, and was sufficient to explain sex differences in social interactions observed in Sprague-Dawley rats (Stack et al., 2010). Indeed, the lower levels of social interaction displayed by females when compared to males are paralleled by lower levels of EGR1 mRNA and proteins in the mPFC. Furthermore, antisense-mediated EGR1 knockdown in the mPFC of males reduced their social interaction levels to those of females (Stack et al., 2010). Conversely, viral-mediated EGR1 overexpression in the mPFC prevents deficits in social interactions induced castration in male rats (Dossat et al., 2017). Similarly, the intracerebroventricular injection of locked-nucleic acid-modified antisense nucleotide knocking down miR-124—which inhibits EGR1—reverses the social interactions impairments in EPAC-KO mice (Yang et al., 2012). Notably, it is particularly interesting to note that partial changes in EGR1 protein levels seen in the studies described above, especially in the mPFC, are sufficient to substantially alter complex behaviors such as social interactions. In addition to indicating that variations in EGR1 levels are critical in determining anxiety levels, this suggests that endogenous variations in EGR1 protein levels in the mPFC such as those occurring throughout the female estrous cycle (Duclot and Kabbaj, 2015) are likely to be associated with variations in anxiety-related behaviors. Accordingly, estrous cycle-dependent variations in anxiety-like behaviors are reported in female rodents (Donner and Lowry, 2013; Barth et al., 2015).

In addition to its association with the development of anxiety- and depression-like states, EGR1 is actively regulated by several classes of antidepressant treatments throughout the brain. While behavioral antidepressant effects are typically observed following chronic, but not acute, treatment, it is surprising to find an up-regulation of EGR1 in the rat hippocampus following a single dose of the tricyclic antidepressant desipramine (Dahmen et al., 1997), or in the rat amygdala following an acute dose of fluoxetine, imipramine, mirtazapine, or lithium chloride (Slattery et al., 2005). Nevertheless, no effects were observed in other brain regions analyzed, suggesting that this effect was relatively constrained (Slattery et al., 2005). Following chronic treatment regimen, the effect is more robust as EGR1 is up-regulated following a wide variety of antidepressant treatments—ranging from the classical antidepressants imipramine and fluoxetine, to electroconvulsive seizures (ECS)—and in multiple key brain areas for antidepressant effects such as the mPFC and hippocampus (Morinobu et al., 1995, 1997; Bjartmar et al., 2000). Interestingly, this effect may not be restricted to neurons as imipramine application on cultured rat astrocytes up-regulates EGR1 expression in a MAPK-dependent manner, which then binds to the glial cell line-derived neurotrophic factor (*gdnf*) gene promoter and activates its expression (Kim et al., 2011). Moreover, while these effects were observed in unstressed systems, which could thus be considered at baseline, experimental interventions exerting an antidepressant-like effect, such as environmental enrichment, FGF2, or fluoxetine, have also been reported to reverse or protect from induction of anxiety- and depressive-like states in multiple models (Monsey et al., 2014; Novaes et al., 2017; Salmaso et al., 2016). Although causality still remains to be clearly established, the up-regulation of EGR1 following antidepressant treatment thus emerges as a key feature of antidepressant response. This is particularly interesting in light of the reduced EGR1 expression found in the frontal cortex of depressed patients who remained symptomatic despite being medicated, as this further suggests that EGR1 up-regulation could represent a reliable marker for positive therapeutic response to antidepressants (Covington et al., 2010).

Interestingly, despite its low expression levels early in the development, EGR1 has been identified as an important mediator of the effects of early-life experience through its transcriptional control of the *gr* gene. For instance, the levels of maternal care received by rat pups during the first week of life determines their neuroendocrine response to stress later in adulthood, through DNA methylation at the hippocampal *gr* promoter located on an EGR1 binding site (Weaver, 2007). As evidence suggests that maternal care triggers serotonin release in the hippocampus, it is particularly interesting that EGR1 knockdown by RNA interference prevents serotonin-induced increase in GR expression in cultured rat hippocampal neurons (Weaver et al., 2007), which thus suggests that the extent of maternal care received by the pup during the first week of life will influence EGR1 binding to the *gr* promoter, which will in turn determine GR expression in a long-lasting manner through epigenetic mechanisms (Weaver, 2007). Notably, children exposed to physical maltreatment—a known risk factor for the development of mood-related alterations in adulthood (Shackman et al., 2007; Shackman and Pollak, 2014)—present with greater DNA methylation of the *gr* promoter, including at the EGR1 binding site (Romens et al., 2015), indicating that such EGR1 control of GR expression by maternal care could also be observed in humans. Moreover, other early-life stressful experiences have similarly been reported to impact EGR1 expression. Maternal separation of C57Bl/6 mice from postnatal day 14–16, for instance, induces a rapid increase in EGR1 expression and its target ARC in the hippocampus through histone acetylation at their respective promoter (Xie et al., 2013). Although causality remains to be determined, these changes are associated with greater dendritic complexity and spine number in the hippocampal CA3 area (Xie et al., 2013), suggesting that early-life experiences can affect neuronal architecture and organization through EGR1. The timing of such manipulation is critical, however, as maternal separation in the same C56Bl/6 strain from postnatal day 2–15 leads to a marked reduction in EGR1 expression in the forebrain neocortex (Navailles et al., 2010).

In addition to shape response to stress later in adulthood, early-life experiences can also impact the development of neuropsychiatric disorders such as schizophrenia. Indeed, adult rats having received high levels of maternal care present with higher GAD1 mRNA hippocampal levels than individuals raised by dams providing low levels of maternal care (Zhang et al., 2010). Notably, this regulation is mediated by EGR1 binding, along with higher H3K9 acetylation and lower DNA methylation, at the *gad1* promoter (Zhang et al., 2010), and thus directly implicates EGR1 in the regulation of GAD1 expression in the brain, which is of particular interest in the context of neuropsychiatric illness in light of the positive correlation between GAD1 and EGR1 expression levels in schizophrenia patients (Kimoto et al., 2014). While the molecular underpinnings of EGR1 alterations in schizophrenia remain unknown, knockdown in cultured hippocampal GABA neurons of the histone deacetylase 1 (HDAC1) and its co-repressor DAXX, whose expressions are also altered in schizophrenia, results in increased GAD1 and *Egr1* mRNA levels, which opens the possibility for an HDAC1/DAXX-mediated repression of EGR1 expression leading to GAD1 inhibition (Subburaju et al., 2016). Furthermore, beyond its etiology, EGR1 is also associated with response to antipsychotic drugs (MacGibbon et al., 1994; Robbins et al., 2008; Bruins Slot et al., 2009; Wheeler et al., 2014; de Bartolomeis et al., 2015), or the psychomimetic phencyclidine in rats (Tamminga et al., 1995; Näkki et al., 1996).

Altogether, the above experimental evidence highlights the important role played by EGR1 in mediating or modulating the stress response and the development of various stress-related disorders. The upstream regulators involved, however, remain unclear and it thus becomes interesting to further consider the link between glucocorticoids released following chronic stress, and EGR1 expression in the central nervous system. Indeed, while EGR1 is a direct regulator of *gr* transcription, activation of GR leads to EGR1 up-regulation in the mouse and rat hippocampus through intracellular signaling pathways involving MAPK (Revest et al., 2005, 2010) or the serum and glucocorticoid regulated kinase 1 (SGK1; Tyan et al., 2008). Notably, the regulation of EGR1 expression by SGK1 involves well-defined mechanisms of *Egr1* transcriptional regulation via the activation by phosphorylation of SRF and CREB, and has been linked to spatial memory formation in rats (Tyan et al., 2008). As its expression in the rodent hippocampus and mPFC rodents is strongly regulated by acute (Bohacek et al., 2015; Mifsud and Reul, 2016) or chronic stress (Anacker et al., 2013; Miyata et al., 2015; Skupio et al., 2015; Cattaneo and Riva, 2016; Wei et al., 2016), SGK1 emerges as a particularly interesting candidate in mediating EGR1 regulations in response to various stress paradigms. In this context, it is particularly interesting to note that SGK1 expression levels are down-regulated in the PFC of post-traumatic stress disorder patients—or increased in the peripheral blood of unmedicated depressed patients (Anacker et al., 2013)—and that SGK1 inhibition in the rat mPFC induces depressive-like behaviors in rodents associated with abnormal dendritic spine morphology and synaptic dysfunction (Licznerski et al., 2015). Altogether, these experimental observations delineate a hypothetical working model in which glucocorticoids release following chronic stress exposure alters SGK1 expression in key brain areas including the hippocampus and mPFC, which in turns regulates *Egr1* transcription through activation of SRF and CREB transcription factors. Although the requirement of EGR1 in SGK1’s effects on neuronal plasticity remains to be determined, EGR1 could in turn orchestrate, through its wide array of targets, the neuronal and synaptic plasticity events underlying the long-term behavioral effects of stress that influence the development of stress-related disorders such as depression or PTSD.

### Drug Reward, Withdrawal and Relapse

Exposure to substance of abuse is a powerful environmental stimulus that triggers a strong neuronal response throughout the brain, but mainly targeting the mesolimbic dopaminergic system, and bears the ability to reorganize existing neuronal connections in a long-lasting manner. IEGs such as EGR1 have thus been repeatedly associated with the neuronal response to large number of compounds with rewarding or addictive properties. EGR1’s involvement in response to cocaine, for instance, are now relatively well-described and reviewed elsewhere (Veyrac et al., 2014). We will thus focus the following section on two distinct classes with rewarding properties.

Opiates, for instance, are known triggers for EGR1 expression in various brain areas. In particular, an acute heroin injection up-regulates *Egr1* mRNA levels in the core and shell of the nucleus accumbens, the dorsal striatum, and the cingulate cortex of C57Bl6 mice (El Rawas et al., 2009). Similarly, increased EGR1 expression levels are observed in the extended amygdala, dorsal striatum, nucleus accumbens shell, and cingulate cortex following an acute morphine injection (Hamlin et al., 2007; Ziółkowska et al., 2012, 2015). Notably, the latter is observed 4 h and 6 h following injection, which suggests that in this context, EGR1 up-regulation is part of a second wave of gene regulations, and is not associated with the rapid hyperlocomotor effects of morphine (Ziółkowska et al., 2015). They do suggest, however, that EGR1 can be involved in neuroadaptations underlying long-lasting effects of morphine exposure, such as withdrawal and relapse. Accordingly, naloxone-induced morphine withdrawal in rats induces an EGR1 up-regulation in the cerebral cortex, hippocampus, thalamus, cerebellum, and brainstem 60 min following the withdrawal (Beckmann et al., 1995). Similarly, EGR1 and its target ARC are up-regulated in the rat dentate gyrus upon morphine-withdrawal memory retrieval which, in light of its established role of in contextual memory reconsolidation in the hippocampus (Lee et al., 2004), suggests that EGR1 could be involved in the synaptic plasticity events underlying reconsolidation of the morphine withdrawal-context (García-Pérez et al., 2016). Under a similar perspective, EGR1 expression is increased in the rat basolateral but not central amygdala during reconsolidation of withdrawal memory, whereas its down-regulation by antisense oligodeoxynucleotides within the basolateral amygdala reduces the withdrawal memory-mediated suppression of heroin seeking (Hellemans et al., 2006), thereby indicating a functional role for EGR1 in encoding heroin seeking in the amygdala. In line with the tight interplay between the amygdala and the mPFC during cue-associated memory reactivation, extinction, or reconsolidation, *Egr1* mRNA levels are also found up-regulated in the rat mPFC following 14 or 30 days of heroin-seeking incubation (Kuntz et al., 2008; Kuntz-Melcavage et al., 2009; Fanous et al., 2013), which thus strengthen further the importance of EGR1 in regulating multiple aspects of opiates dependance.

Similar to opiates, alcohol consumption triggers a marked EGR1 response throughout the brain. In adult rats and mice, acute ethanol exposure leads to increased EGR1 expression in the mPFC, central amygdala, medial amygdala, supraoptic nucleus, PBN, lateral part of the caudate putamen, prelimbic and infralimbic cortices, orbitofrontal cortex, hippocampus and nucleus accumbens (Thiriet et al., 2000; Faria et al., 2008; Hansson et al., 2008; Lindholm et al., 2008; Liu and Crews, 2015). Repeated exposure for 15 days, however, or chronic intermittent exposure, reduces *Egr1* mRNA levels in the mPFC, hippocampus, and nucleus accumbens (Repunte-Canonigo et al., 2007; Faria et al., 2008), which thus indicates that EGR1’s regulation by ethanol depends on the nature and duration of the exposure. Moreover, this dynamic regulation of EGR1 by ethanol is further illustrated upon withdrawal. Indeed, *Egr1* mRNA and protein levels are up-regulated in the cerebral cortex, olfactory bulb, inferior colliculus, brainstem, and hippocampus of ethanol-dependent rats at 12 h and 15–24 h, respectively, following withdrawal (Matsumoto et al., 1993), a process associated with increased EGR1 DNA binding activities in the cerebral cortex from 16 h to 72 h following withdrawal (Beckmann et al., 1997). Interestingly, while withdrawal-induced anxiety-like behaviors emerge within the same period, between 8 h and 17 h following withdrawal (Matsumoto et al., 1993), withdrawal-induced anxiety-like behaviors in mice, which rise on the 1st day of withdrawal and last up to 21 days later, are positively correlated with the increase in EGR1-positive cells in the central amygdala and bed nucleus of the stria terminalis (Lee et al., 2015), which thus link the increase in EGR1 expression in the amygdala to the development of anxiety-like symptoms upon ethanol withdrawal.

## Conclusions

In this review article, we summarized and discussed the regulations and functions of the IEG EGR1 in the central nervous system relevant to neuropsychiatric disorders. Situated downstream of general signaling pathways activated by neuronal activity, EGR1 has been found regulated by a wide variety of environmental events that position EGR1 as a critical integrator and mediator of environmental influences on neuronal activity. Furthermore, due to its very large range of potential transcriptional targets identified so far, the reach of EGR1’s functions in neurons continues to expand. In particular, without considering eventual indirect effectors, EGR1 can alter the expression of genes related to every level of synaptic plasticity, from vesicular transport and release of neurotransmitters, to synaptic architecture, endocytosis, and protein degradation (Figure 2). Notably, in line with its sex- and estrous cycle-dependent expression in the rat mPFC, it is important to consider that this control of synaptic plasticity by EGR1 is likely to substantially vary between sexes in an estrous cycle-dependent manner. Despite such wide array of synaptic plasticity-related potential targets and its well-known association with neuronal activity. however, the current knowledge of the precise mechanisms by which EGR1 influences synaptic and neuronal plasticity, as well as the direct targets involved, remains paradoxically unclear and requires to be clearly described and validated *in vivo*. Nevertheless, EGR1 is tightly associated to neuronal activity throughout the brain and can thus be used as a reliable tool for mapping neuronal activity in response to a given environmental event. In this context, it is possible to consider that a substantial amount of EGR1’s regulations described in this review simply reflect neuronal responses in a given structure to a given behavioral stimulation. It is important to note, however, that EGR1 governs specific neuronal processes, which can be reflected, for instance, by its specific involvement in the maintenance but not induction of LTP, or, under appropriate conditions, memory reconsolidation but not acquisition, for instance. In line with its crucial role in shaping neuronal response, EGR1 is associated with the etiology and treatment of most common neuropsychiatric disorders such as major depressive disorder, anxiety disorders, schizophrenia, or addiction. Therefore, despite its widespread mode of regulation, EGR1 functions in the central nervous system are complex and represent a valuable candidate for investigating gene × environment interactions.

## Author Contributions

FD and MK participated equally in the article design and outline; FD then wrote the first draft. After a few revisions and editing by both authors, the article was submitted.

## Funding

This work was supported by grants from the National Institute of Mental Health (NIMH) MHR01 MH87583, MH099085 and MH109450 to MK.

## Conflict of Interest Statement

The authors declare that the research was conducted in the absence of any commercial or financial relationships that could be construed as a potential conflict of interest.
